# Altered Blood Flow Response to Small Muscle Mass Exercise in Cancer Survivors Treated With Adjuvant Therapy

**DOI:** 10.1161/JAHA.116.004784

**Published:** 2017-02-08

**Authors:** Kaylin D. Didier, Austin K. Ederer, Landon K. Reiter, Michael Brown, Rachel Hardy, Jacob Caldwell, Christopher Black, Michael G. Bemben, Carl J. Ade

**Affiliations:** ^1^ Department of Health and Exercise Science The University of Oklahoma Norman OK; ^2^ Department of Kinesiology Kansas State University Manhattan KS

**Keywords:** arterial pressure, blood flow, cancer, exercise, exercise physiology, Exercise, Physiology

## Abstract

**Background:**

Adjuvant cancer treatments have been shown to decrease cardiac function. In addition to changes in cardiovascular risk, there are several additional functional consequences including decreases in exercise capacity and increased incidence of cancer‐related fatigue. However, the effects of adjuvant cancer treatment on peripheral vascular function during exercise in cancer survivors have not been well documented. We investigated the vascular responses to exercise in cancer survivors previously treated with adjuvant cancer therapies.

**Methods and Results:**

Peripheral vascular responses were investigated in 11 cancer survivors previously treated with adjuvant cancer therapies (age 58±6 years, 34±30 months from diagnosis) and 9 healthy controls group matched for age, sex, and maximal voluntary contraction. A dynamic handgrip exercise test at 20% maximal voluntary contraction was performed with simultaneous measurements of forearm blood flow and mean arterial pressure. Forearm vascular conductance was calculated from forearm blood flow and mean arterial pressure. Left ventricular ejection time index (LVETi) was derived from the arterial pressure wave form. Forearm blood flow was attenuated in cancer therapies compared to control at 20% maximal voluntary contraction (189.8±53.8 vs 247.9±80.3 mL·min^−1^, respectively). Forearm vascular conductance was not different between groups at rest or during exercise. Mean arterial pressure response to exercise was attenuated in cancer therapies compared to controls (107.8±10.8 vs 119.2±16.2 mm Hg). LEVTi was lower in cancer therapies compared to controls.

**Conclusions:**

These data suggest an attenuated exercise blood flow response in cancer survivors ≈34 months following adjuvant cancer therapy that may be attributed to an attenuated increase in mean arterial pressure.

## Introduction

Chemotherapy or a combination of chemotherapy and radiation are frequently used to treat several types of cancer. Cancer survivors treated with these anticancer therapies have an increased survival rate,^1^ but are often at an increased risk for cardiovascular disease, with myocardial injury consistently reported as a long‐term treatment side effect (for review, see Khouri et al^2^). However, the cardiovascular toxicity observed within the myocardium may also occur within the peripheral vasculature, given that the mean vascular age of cancer survivors has been reported to be ≈8 years greater than chronological age, suggesting that a degree of vascular dysfunction may be present within the vasculature of some cancer survivors.^3^ Despite the increased risk of cardiovascular disease, established cardiac toxicity, and advanced vascular aging in many cancer survivors, the effects of chemotherapy and radiation on vascular function remains poorly understood, particularly in the years following treatment.^4^

To date, the effects of chemotherapy and radiation on vascular health have primarily been studied in childhood cancer survivors or following a single treatment session.^5^, ^6^, ^7^ Chow et al observed a decreased brachial artery flow‐mediated dilation (FMD), a measurement of endothelial‐dependent dilation, ≈20 months following anthracycline‐based chemotherapy.^5^ Similarly, Vaughn et al demonstrated a decreased FMD in long‐term survivors of testicular cancer. These decreases in vascular function, which are associated with increased mortality and morbidity, can lead to decreases in blood flow and increases in arterial stiffness.^8^ As such, in subjects receiving anthracycline chemotherapy, Chaosuwannaki et al, Miza‐Stec et al, and Draft et al independently demonstrated significant increases in aortic stiffness 4 to 6 months following treatment, which, attributed to increase in vascular resistance, can decrease peripheral blood flow.^9^, ^10^, ^11^, ^12^

Decreases in limb blood flow and vascular conductance have been shown to have key implications in both function and disease risk in humans.^13^, ^14^ Whereas whole‐body exercise stress is commonly used in the clinical setting to evaluate integrated cardiorespiratory function, it is limited by an inability to investigate key peripheral vascular responses. Handgrip exercise is a common exercise model that utilizes a small muscle mass and allows for direct noninvasive investigation of blood flow control.^15^, ^16^ Importantly, decreases in vascular function and the control of blood flow during exercise will decrease exercise capacity, which is inversely related to mortality rate in healthy individuals^17^, ^18^ and may play a role in the development and persistence of cancer‐related fatigue.^19^ Given that little is known to date about the peripheral vascular control of blood flow in cancer survivors, the purpose of the present study was to investigate vascular responses to exercise in cancer survivors previously treated with adjuvant cancer therapies. It was hypothesized that in relation to healthy cancer‐free controls, cancer survivors previously treated with chemotherapy or a combination of chemotherapy and radiation would have a significantly decreased limb blood flow during submaximal dynamic forearm exercise. To test these hypotheses, exercise blood flow was measured by two‐dimensional (2D) Doppler ultrasound during dynamic handgrip exercise. This modality of exercise was utilized because it allows for controlled workloads and stable noninvasive vascular measurements compared to traditional lower‐limb treadmill or cycling exercise. As such, this modality of exercise has previously been used to evaluate exercise blood flow responses in aged and clinical populations.^15^, ^16^

## Methods

### Subjects

This study utilized a cross‐sectional, case‐control study design, with 11 participants recruited to a cancer survivor group (10 women) and 9 participants recruited to a control group (8 women). Control participants were selected if their age and maximal voluntary contraction (MVC) were within 2.5 SDs of the means for the cancer survivor group. All women were postmenopausal. History of chemotherapy and radiation treatment history, date of last treatment, and confirmation of ≥12 months from cancer diagnosis was obtained from the treating oncologists or family practitioner for all cancer survivor participants. Individuals in the control group were recruited from local advertisements. Exclusion criteria included previous diagnosis with diabetes mellitus, cystic fibrosis, cardiopulmonary disease, chronic obstructive pulmonary disease, asthma, lung disease, or microvascular/peripheral artery disease or were currently anemic as determined by health history questionnaire. Subjects were free of signs or symptoms indicating the presence of pulmonary, metabolic, or cardiovascular disease. Individuals with uncontrolled hypertension (systolic blood pressure [SBP], >160 mm Hg), or currently smoking were excluded from the study. None of the participants were currently taking prescription blood pressure medication or other prescribed medication. Current level of physical activity was determined using the International Physical Activity Questionnaire.^20^ Informed consent was obtained from all individuals included in the study according to the University of Oklahoma (Norman, OK) Institutional Review Board for Research Involving Human Subjects requirements. All testing was conducted in a temperature‐controlled laboratory (21–23°C) after a 4‐hour fast and having refrained from strenuous exercise, alcohol, and caffeine for at least 12 hours.

### Experimental Protocol

MVC was measured in triplicate in the right arm with a handgrip dynamometer (EH101; Camry, South El Monte, CA) and highest value recorded. Following a 10‐minute rest supine period, single‐arm dynamic handgrip exercise was performed in the right arm on a custom‐built handgrip ergometer with the arm abducted to ≈80° at heart level. Each participant performed a workload equivalent to 20% MVC for 4 minutes to ensure a steady state was achieved. Given that forearm blood flow (FBF) is related to the absolute workload performed^21^ and potential arterial occlusion occurs with ≥30% MVC workloads,^22^ cancer survivor and control participants were group matched for MVC so that both absolute and relative exercise workloads would be similar between groups. Each workload was achieved by lifting the prescribed weight ≈3 cm over a pulley at a duty cycle of 1 second contraction and 2 seconds relaxation, with a metronome used to ensure correct timing. To minimize the potential risk of external stimuli altering cardiovascular responses, participants were asked to refrain from talking and to remain as still as possible while the experimental measurements were performed throughout baseline and exercise study periods.

### Experimental Measurements

Resting mean arterial blood pressure (MAP) was measured from the average of 3 brachial artery pressure recordings following a 10‐minute rest period in the supine position (Omron 10 Plus Series; Omron Healthcare, Hoofddorp, The Netherlands). Baseline and handgrip exercise beat‐by‐beat MAP was continuously measured by calibrated finger photoplethysmography (Finometer Pro; Finapress Medical Systems, Amsterdam, The Netherlands) and averaged over the last 30 seconds of exercise. This system has previously been used to evaluate blood pressure responses to handgrip exercise in clinical populations.^23^ In all instances, arterial pressure recordings were performed at heart level. In addition, the data for each beat were processed to obtain measurements of left ventricular ejection time (LVET), which was corrected for changes in heart rate to determine LVET index (men, LVETi 1.7×heart rate+LVET; women, LVETi 1.6×heart rate+LVET).^24^ Arterial pressure wave‐form–derived measurements of LVETi can be affected by changes in ventricular performance and vascular tone (eg, elastiance, compliance, and stiffness), which may lead to over‐ or underestimation of measurements obtained at the left ventricle and therefore should be interpreted only as an estimate of ventricular function.

A 2D and Doppler ultrasound system (Logiq S8; GE Medical Systems, Milwaukee, WI) with a linear array transducer operated at an image frequency of 10.0 MHz was used to simultaneously measure the brachial artery diameter and blood velocities. Doppler velocity measurements were made in pulse‐wave mode at a Doppler frequency of 4.0 MHz and corrected for an angle of insonation less than 60 degrees. During handgrip exercise, all ultrasound measurements were made 5 to 10 cm above the antecubital fossa, midway between the antecubital and axillary regions, medial to the *m. biceps brachii*. Time‐averaged mean velocity values were measured and averaged over a 3‐second interval on the ultrasound system using the manufacturer's onscreen software. Brachial artery diameter was calculated at 15 Hz and averaged into 3‐second bins using an automated edge‐detection software (Medical Imaging Applications, Coralville, IA).

During the fourth minute of the handgrip exercise, workload 2D ultrasound images and Doppler velocity waveforms were recorded with the last 30 seconds used for data analysis. Time‐aligned artery diameter and mean velocity were used to calculate FBF [forearm blood flow (mL·min^−1^)=mean velocity×π×vessel radius^2^×60]. Forearm vascular conductance (FVC) [mL·min^−1^·(100 mm Hg)^−1^] was calculated as the ratio of FBF to the time‐aligned MAP [forearm vascular conductance (mL·min^−1^·(100 mm Hg)^−1^)=(forearm blood flow/mean arterial pressure)×100].

### Statistical Analysis

All statistical tests were conducted using a commercial statistical software package (SigmaPlot/SigmaStat 12.5; Systat Software, Point Richmond, CA). Subject characteristics were analyzed with parametric or nonparametric statistics following a Shapiro–Wilk test of normality. Cardiovascular responses with exercise were analyzed by a 2‐factor, repeated‐measures ANOVA with Student–Newman–Keuls post‐hoc analyzes. Given the large post‐treatment time frame within the cancer survivor group, linear regression analysis was used to evaluate the relationship between months post‐treatment and FBF response. Statistical significance was declared when *P*<0.05. All data are presented as mean±SD, unless otherwise stated.

## Results

Individual cancer survivor characteristics of age, sex, months since last date of treatment, chemotherapy drugs used, and radiation exposure are presented in Table 1. Table 2 describes the baseline characteristics of each group. There were no significant differences in age, height, weight, and body mass index (BMI) between cancer survivor and control participants. Resting SBP, diastolic blood pressure (DBP), and MAP were also not different between groups (*P*<0.05). Forearm MVC and the absolute workload performed at 20% MVC were not different between groups. Thus, participants were performing both the same absolute and relative exercise workloads. In the cancer survivor group, 9 (82%) individuals were classified as inactive and 2 (18%) as minimally active individuals. The control group was composed of 6 (67%) inactive and 3 (33%) minimally active individuals.

**Table 1:** Cancer Survivor Treatment Characteristics

ID No.	Age (y)	Sex	Cancer Type	Months Post‐Treatment	Chemotherapy Treatment	Radiation Treatment
1	55	F	Breast cancer	41	Cytoxan+taxol	Yes
2	54	F	Breast cancer	35	Adriamyacin+cytoxan+ taxotere+abraxane	Yes
3	59	F	Breast cancer	121	Taxotere+neupogen	No
4	56	F	Breast cancer	40	Herceptin+taxotere+carboplatin+fermara	No
5	59	M	Lymphoma	31	Cytoxan+adriamycin+vincristine+etoposide prednisone+ifosamide+caroplatin	Yes
6	52	F	Breast cancer	36	Taxotere+cytoxan	Yes
7	67	F	Breast cancer	22	Taxotere+cytoxan+neulasta	No
8	69	F	Breast cancer	12	Arimidex+aromasin	Yes
9	52	F	Breast cancer	13	Adriamycin+cytoxan+taxol	Yes
10	52	F	Breast cancer	9	Adriamycin+cytoxan+methotrexate+fluorouracil+tamoxifen+taxotere+perjeta+herceptin+faslodex+arimidex	Yes
11	62	F	Breast cancer	21	Tamoxifen	Yes

**Table 2:** Participant Characteristics

Characteristics	Value
Control participants	
Sex (M/F)	1/8
Age, y	56±6
Height, cm	166.7±6.0
Weight, kg	72.1±17.7
BMI, kg/m^2^	25.5±6.5
MVC, kg	30±8
Heart rate, bmp	61±9
SBP, mm Hg	148±23
DBP, mm Hg	78±6.0
Cancer subjects	
Sex (M/F)	1/10
Age, yr	58±6
Height, cm	167.0±4.5
Weight, kg	79.9±19.54
BMI, kg/m^2^	28.6±6.3
MVC, kg	28±8
Heart rate, bpm	67±6
SBP, mm Hg	150±22
DBP, mm Hg	76±11

Values expressed as means±SD. BMI indicates body mass index; bpm, beats per minute; DBP, diastolic blood pressure; MVC, maximal voluntary contraction; SBP, systolic blood pressure.

**Figure 1:**
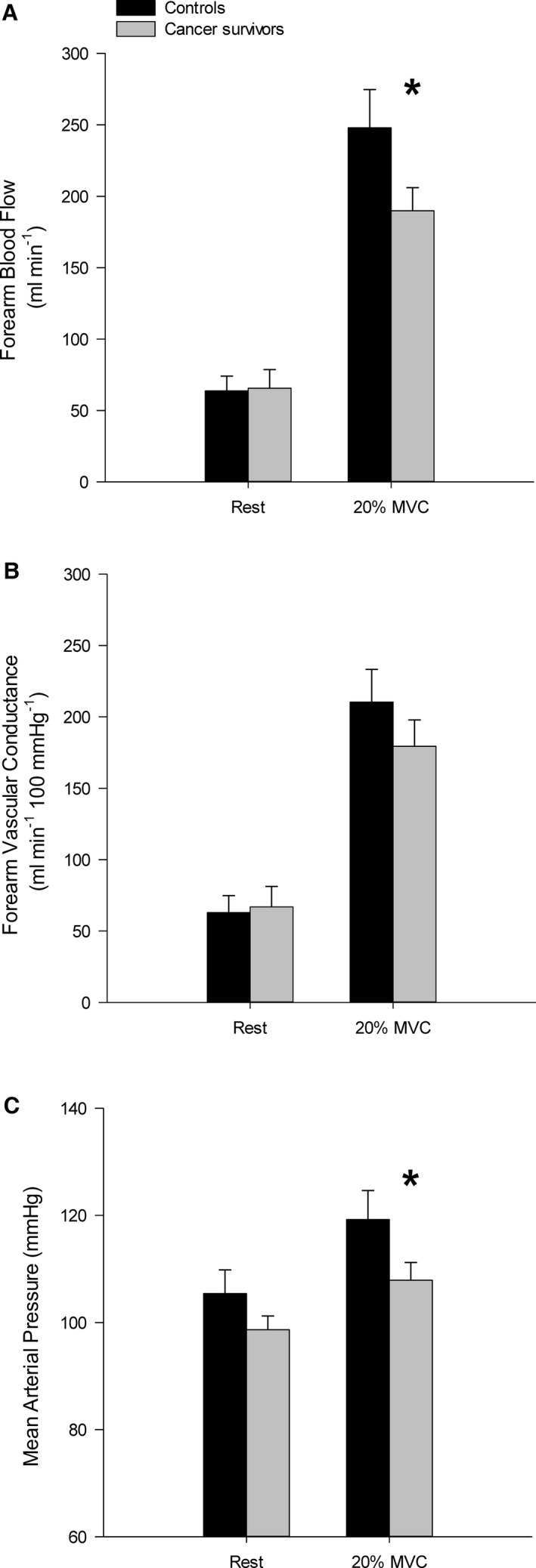
Forearm blood flow (A), forearm vascular conductance (B), and mean arterial pressure (C) responses during dynamic forearm exercise at 20% maximal voluntary contraction (MVC). Forearm blood flow response was significantly decreased in cancer survivors compared to controls. Mean arterial pressure response was lower in cancer survivors during exercise compared to controls, but not at rest. *P<0.05 versus control. Mean±SE.

**Figure 2:**
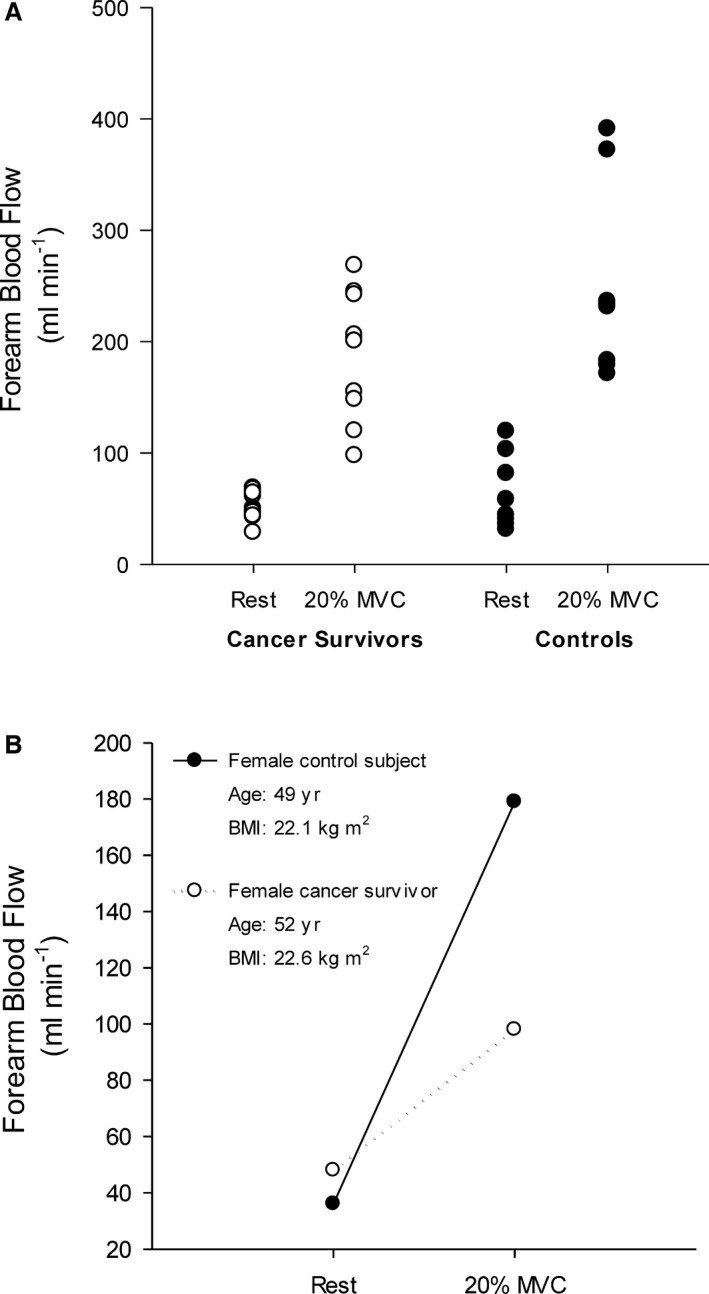
Forearm blood flow response to handgrip exercise in individual cancer survivors and controls (A). Given that control participants were selected if their age and maximal voluntary contraction (MVC) were within 2.5 SDs of the means for the cancer survivor group, a representative female cancer survivor and control are also presented who were similar in both age and BMI (B). BMI indicates body mass index.

**Figure 3:**
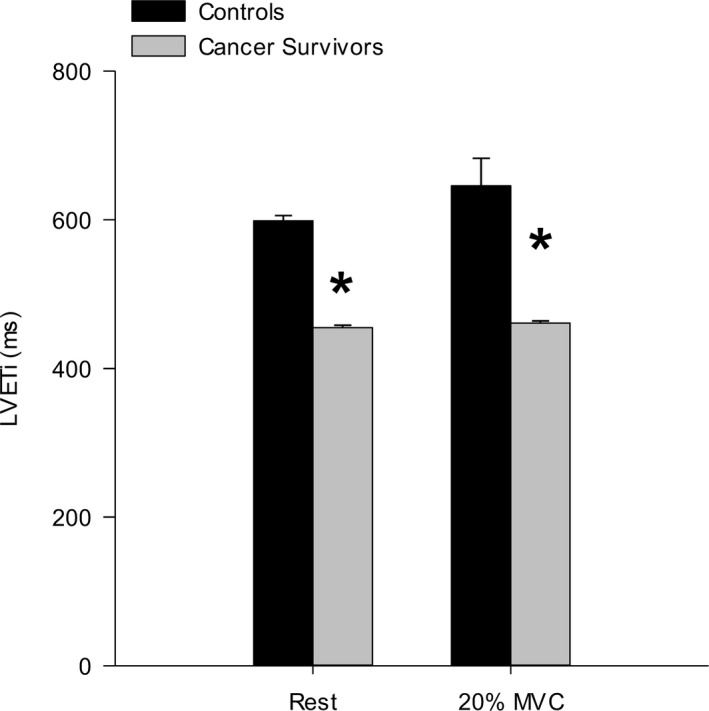
Left ventricular ejection time index (LVETi) derived from the arterial pressure waveform during dynamic forearm exercise at 20% maximal voluntary contraction (MVC). LVETi was significantly decreased in cancer survivors compared to controls. *P<0.05 versus control. Mean±SE.

Supine, resting FBF was not different between cancer survivors and controls (survivors, 63.7±41.3; controls, 63.0±31.2 mL·min^−1^; *P*=0.97). During handgrip exercise at 20% MVC, FBF was significantly lower in the cancer survivors compared to controls (Figure 1A). Figure 2 illustrates FBF response in a representative cancer survivor and control participant. Note that FBF is lower in the cancer survivor despite performing a similar absolute workload. The association between the number of months post‐treatment and FBF response was not statistically significant (*r*=0.5; SEE, 42.77; *P*=0.13). FVC was not different between cancer survivors and controls during the exercise trial (*P*=0.15; Figure 1B). MAP was significantly decreased in the cancer survivors during exercise compared to the control group (Figure 1C). In addition, the estimate of LVETi from the arterial pulse wave was significantly lower in the cancer survivors compared to controls at rest and during the exercise trail (Figure 3).

## Discussion

This study has provided new insight into the peripheral cardiovascular responses to dynamic handgrip exercise in long‐term cancer survivors previously treated with adjuvant cancer therapies. First, FBF was attenuated in the cancer survivors during moderate‐intensity handgrip exercise, but not FVC. Second, in this group of cancer survivors, arterial pressure response to exercise was attenuated, which may have contributed to the attenuated response in FBF during exercise observed in cancer survivors. Taken together, these reveal that a cancer survival‐related attenuation of forearm muscle blood flow during exercise may not be a consequence of differences in vascular conductance, but may be related to alterations in blood pressure control.

The present findings indicate that the steady‐state FBF response in the cancer survivors is attenuated during moderate‐intensity handgrip exercise (Figure 1A). This difference was not attributed to differences in the absolute workloads performed by each group given that the groups were matched for MVC so that both the same absolute and relative exercise workloads were performed. Moreover, as can be seen in Figure 2, a representative cancer survivor had an attenuated FBF response compared to a control participant matched for age, sex, and with an identical MVC, which reflects the mean responses illustrated in Figure 1. Furthermore, we believe the differences observed between groups is attributed to an reduced blood flow response in the cancer survivors, not an increased response in our controls, given that our steady‐state blood flow and vascular conductance values reported for the present study's control participants is similar to that reported for healthy aging.^16^

Our investigation into the underlying factors dictating the observed decrease in FBF in the cancer survivors revealed that FVC was not different compared to healthy controls. The unaltered vascular conductance observed in the cancer survivors is somewhat at odds with some reports of cardiovascular function in the weeks following exposure to cancer therapies. Several studies have examined the effects of chemotherapy treatments on cardiovascular health^25^ and resting cardiac^2^, ^26^ and peripheral vascular^4^ function. To date, several investigations have demonstrated increases in arterial stiffness within a few weeks following anthracycline chemotherapy compared to age‐matched controls.^10^, ^11^, ^12^ Similarly, acute exposure to chemotherapy reduces endothelium‐dependent vasodilation.^6^ Previous studies in testicular cancer survivors and childhood survivors of leukemia suggest that endothelium‐dependent dilation is decreased on the order of 50% to 75% compared control populations.^5^, ^27^ In contrast to these studies, Jones et al reported no difference in endothelium‐dependent FMD in adult breast cancer survivors ≈20 months post‐treatment compared to healthy controls.^28^

During exercise, the increase in FVC in the active arm occurs through the integration of mechanical factors (ie, muscle pump) and locally derived endothelial and metabolic substances.^29^ There is limited evidence that acute exposure to radiation or chemotherapy negatively impacts these factors. Several studies report altered smooth muscle sensitivity to vasoactive substances, including nitric oxide, following radiation exposure^30^, ^31^ and nitric oxide bioavailability may also be decreased following radiation as a consequence of an increased oxidative stress.^31^ Similarly, Duquaine et al report significantly decreased nitrite and nitrate concentrations, which is an indirect index of nitric oxide production, in patients after a single dose of chemotherapy.^6^ Endothelial injury has also been reported following exposure to doxorubicin chemotherapy in organ culture and animal models.^32^, ^33^ In the initial months following anthracycline chemotherapy in pediatric cancer patients, Chow et al observed a decreased brachial artery endothelium‐dependent vasodilation.^5^ Similarly, in women survivors of breast cancer, Beckman et al observed a decrease endothelium‐dependent vasodilation in arteries directly exposed to external‐beam radiation therapy compared to healthy women.^34^ However, no endothelial dysfunction was observed in the nonirradiated arteries of the same women, suggesting that the negative cardiovascular effects of radiation occur only in exposed regions.

The increase in FVC above baseline during exercise in the cancer survivors of the present study could be taken to suggest that the observed decreases in endothelial function following acute radiation and chemotherapy in other studies does not persist long term. This conclusion is supported by the work of Jones et al and Ederer et al, who demonstrated no difference in brachial artery endothelium‐dependent FMD in cancer survivors compared to healthy controls.^28^, ^35^ Given the potential role several factors may play in mediating changes in vascular conductance during dynamic exercise, future investigations will need to determine the relative roles factors like nitric oxide and prostacyclin have during exercise in cancer survivors. Additional investigations will also be required to determine the role vasodilator pathways, particularly those related to endothelial function, have in mediating blood flow during heavy and severe exercise intensities.

Limb blood flow during dynamic exercise is dictated not only by local increases in vascular conductance, but also by increases in perfusion pressure through increases in MAP. This increase in MAP is coordinated by integration of adjustments in cardiac output, the exercise pressor reflex, arterial baroreflex, and central command.^36^ Of interest in the present study is the observed difference in MAP during exercise in cancer survivors compared to healthy controls. Evaluation of the blood pressor responses to handgrip exercise in human clinical and aging populations compared to healthy young controls regularly utilizes relative exercise intensities.^37^ However, it is important to recognize that the MAP response to exercise is workload and muscle mass dependent.^38^ Therefore, our goal was to match the control participants by both age and MVC to the cancer survivor group, which resulted in an absolute workload performed by each group that was not statistically different. Therefore, other factors may have played a key role in the attenuated MAP response observed in the cancer survivors compared to healthy controls.

Regarding possible mechanism, decreases in cardiac output may have contributed to the decreased FBF and MAP response in the cancer survivor group. Whereas handgrip exercise only requires a fraction of maximal cardiac output, a significant relationship exists between cardiac output and limb blood flow at rest.^39^ In clinical cardio‐oncology practice, an asymptomatic decrease in left ventricular ejection fraction (LVEF) is the most commonly observed form of cardiotoxicity.^40^ LVEF deterioration has been shown to occur in cancer survivors within the first year of exposure to chemotherapy treatment and can continue to decrease in the months following chemotherapy treatment. In addition, several reports have demonstrated a decreased LVEF during exercise following cancer treatment with doxorubicin.^41^, ^42^ During exercise, increases in cardiac output are achieved, in part, through increase in stroke volume attributed to increases in ventricular preload and contractility. Therefore, if LVEF was reduced in the cancer survivors, it is possible that it played a role in limiting the increase in MAP and subsequent limb perfusion pressure and blood flow. In the present study, direct measurements of cardiac function were not made and limit our ability to mechanistically determine the factors contributing the decreased limb blood flow in the cancer survivor group. However, estimates of LVETi derived from the arterial pressure waveform were significantly decreased in the cancer survivors compared to controls. These data suggest that decreases in left ventricular performance may have contributed to the decreased FBF observed in the cancer survivors. However, this should be interpreted with caution given that estimates of LEVT from peripheral arteries may not completely reflect what occurs at the left ventricle. Future investigations performing simultaneous cardiac and vascular evaluations during exercise stress are required to fully address this issue.

The increase in arterial pressure during dynamic exercise is also achieved by adjustments within the autonomic nervous system through the integration of central command, arterial baroreflex, and the exercise pressor reflex.^43^ Several studies to date have pointed to the presence of chemotherapy‐induced peripheral neuropathy in cancer patients receiving adjuvant therapy, which often does not completely recover (for review, see Quasthoff and Hartung^44^). Given the integral role of the type III and IV afferent arms of the exercise pressor reflex during exercise in mediating the mechanically and chemically evoked increases in MAP,^43^ it is possible that some form of peripheral neuropathy still existed in the present study's cancer group. In addition, an attenuated increase sympathetic nerve activity subsequent to a depressed exercise pressor reflex would have an adverse impact on cardiac function. Peripheral neuropathy is commonly assessed in cancer patients using a series of questionnaires or by clinician grading.^45^ Unfortunately, an assessment of peripheral neuropathy was not performed in the present study and will require further investigation to determine its role in blood pressure control during exercise.

### Implications

In addition to cardiotoxicity and changes in cardiovascular disease risk, cancer survivors have a decreased exercise capacity^46^ and presence of cancer‐related fatigue symptoms.^47^, ^48^ Of importance, both have been shown to be related to mortality rate^17^, ^18^ and functional outcomes (eg, ability to return to work).^49^ In many patient populations, like heart failure, the peripheral blood flow response to exercise is a key mechanism in determining exercise capacity and could play a key role in cancer patients.^50^, ^51^ Although this study did not evaluate exercise capacity or cancer‐related fatigue, it is reasonable to speculate that changes in peripheral vascular function may be an underlying factor in the decreased exercise capacity associated with adjuvant cancer therapies and in cancer survivors.^51^ To date, no other studies have investigated peripheral blood flow responses to exercise in cancer survivors, and the present findings suggest that peripheral cardiotoxicity with cancer treatments should not be overlooked.

### Limitations

The strengths of the study include the use of a diverse group of cancer survivors, which allow the findings to be generalized to more than a single chemotherapy drug, radiation exposure, or cancer type. Similar to the present study, other studies have also used participants from different cancer populations when evaluating evidence of cardiovascular disease.^11^ However, having a diverse group of cancer populations is a limitation and does not allow the exact treatment‐related mechanisms to be explained. For instance, it is well established that anthracycline chemotherapy drugs negatively impact long‐term cardiovascular health.^2^ Additionally, the taxane class of chemotherapy drugs are often associated with chemotherapy‐induced peripheral neuropathy.^52^ As noted in Table 1, the cancer survivors in the present study were treated with various combinations of anthracycline and taxane class chemotherapies. Whereas certain types of chemotherapy medications, like anthracycline chemotherapy, are associated with adverse cardiac health, there is a paucity of information regarding the effects of specific chemotherapies on peripheral vascular health. Given this, we had no justification for examining only 1 type of chemotherapy or cancer at this time. Therefore, the type of chemotherapy and type of cancer were not controlled for, but was recorded for each participant. There are several additional limitations to this study that need to be addressed. First, the population studied was predominantly female and therefore does not accurately represent all cancer survivors.^53^ Second, the sample size was modest, but very similar to previous investigations investigating the effects of healthy aging on the peripheral cardiovascular responses to dynamic handgrip exercise.^16^, ^54^ The present study also did not evaluate differences in arterial stiffness because of a lack of proper equipment at the time of data collection. The fourth limitation concerns the cross‐sectional design. A longitudinal study design would have been more robust and may have allowed for a more‐mechanistic evaluation of blood flow and vasodilator responses following adjuvant cancer treatments. This type of study design would have minimized the effects of various confounding variables that may have introduced variability into the present study.

### Summary

This is the first study, to our knowledge, to quantify and characterize the impact of previous adjuvant cancer therapy in cancer survivors on peripheral vascular responses to dynamic exercise. During dynamic handgrip exercise in cancer survivors previously treated with adjuvant cancer therapy, an attenuated blood flow and arterial pressure responses were observed compared to controls. In contrast, FVC during exercise was not influenced by cancer survival. These findings highlight the contribution the decreased arterial pressure response to exercise may have on attenuated blood flow response in our cancer survivors. The mechanistic reasons for these findings and the functional consequences can only be speculated on at this point, but may, in total, contribute to the altered cardiorespiratory function previously reported in this population.^46^

## Sources of Funding

Publication of this article was funded in part by the Kansas State University Open Access Publishing Fund.

## Disclosures

None.
